# Circadian Deregulation: Back Facing the Sun Toward Metabolic Dysfunction-Associated Steatotic Liver Disease (MASLD) Development

**DOI:** 10.3390/nu16244294

**Published:** 2024-12-12

**Authors:** Mariana Verdelho Machado

**Affiliations:** 1 Gastroenterology Department, Hospital de Vila Franca de Xira, 2600-009 Vila Franca de Xira, Portugal; mverdelhomachado@gmail.com; Tel.: +351-912620306; 2 Clínica Universitária de Gastrenterologia, Faculdade de Medicina, Universidade de Lisboa, Avenida Prof. Egas Moniz, 1649-028 Lisboa, Portugal

**Keywords:** circadian clocks, MASLD, hepatocellular carcinoma, chrononutrition

## Abstract

Earth’s rotation around its axis has pressured its inhabitants to adapt to 24 h cycles of day and night. Humans adapted their own circadian rhythms to the Earth’s rhythms with a light-aligned awake–sleep cycle. As a consequence, metabolism undergoes drastic changes throughout the circadian cycle and needs plasticity to cope with opposing conditions in the day (when there is an increase in energy demands and food availability), and during the night (when prolonged fasting couples with cyclic changes in the energy demands across the sleep stages). In the last century, human behavior changed dramatically with a disregard for the natural circadian cycles. This misalignment in sleep and eating schedules strongly modulates the metabolism and energy homeostasis, favoring the development of obesity, metabolic syndrome, and metabolic dysfunction-associated steatotic liver disease (MASLD). This review summarizes the effects of circadian disruption, with a particular focus on the feeding and sleep cycles in the development of MASLD and hepatocellular carcinoma.

## 1. Introduction

Obesity has been declared a growing global epidemic by the World Health Organization (WHO), with one in eight adults being obese (and two in five overweight) worldwide, which represents a two-fold increase in its prevalence in the last 30 years [1]. Obesity-associated conditions are also on the rise, particularly insulin resistance, type 2 diabetes mellitus, and the metabolic syndrome [2]. Those statistics explain the global epidemic of metabolic dysfunction-associated steatotic liver disease (MASLD, formerly known as nonalcoholic fatty liver disease) that currently afflicts up to one-third of the population, which represents a 50% increase in the last 30 years [3].

The rapid rise in obesity-related conditions and MASLD probably reflects a rapid shift in the environment and in collective behavior, and could probably be mitigated by non-pharmacological approaches. Indeed, 30–80% of the risk of MASLD development cannot be explained by heritability [4].

Life has evolved by adapting to the 24 h day–night cycle resulting from the Earth’s rotation around its axis [5,6]. To cope with these cyclic environmental changes, organisms synchronize behavior (sleep–awake cycle, fasting–feeding cycle), and physiological functions, including metabolic functions, in response to external cues, the zeitgebers (from the German for “time givers”), through circadian clocks. The term circadian rhythm derives from the Latin term *circa diem* (meaning “around one day”), and was first proposed by Franz Halberg in 1959 [7].

Human lifestyle has evolved with a disregard for the natural cyclic environmental cues, with frequent misalignment in our behavior, such as sleep–awake and fasting–feeding cycles, with the circadian clocks. For example, artificial light allows us to choose the time to sleep, jet lag and night shift work impose different sleep patterns, and the timing of food ingestion is mainly determined by convenience.

Recent accumulated evidence suggests that circadian misalignment is associated with metabolic deregulation and disease, namely obesity [8], type 2 diabetes mellitus [9], metabolic syndrome [10], MASLD, and progression to hepatocellular carcinoma [11,12].

This review will critically summarize the physiopathology of circadian rhythms and how its deregulation may promote the development of MASLD and hepatocellular carcinoma, with particular emphasis on the clinical consequences of chrononutrition and sleeping behaviors.

## 2. Circadian Regulation

The circadian rhythm is regulated by a hierarchical system, organized by a central clock synchronized with peripheral clocks. The dominant clock is localized in the suprachiasmatic nuclei of the anterior hypothalamus and sends neuronal, hormonal, and metabolic signals to synchronize with the peripheral clocks [13]. All organs and virtually all cells have internal clocks that, even though they are under the umbrella of the central clock, are autonomous and self-sustained [6,14]. The central clock responds primarily to the light, which is perceived by the photoreceptive retinal ganglion cell (ipRGC) expressing the photopigment melanopsin and connects with the central clock through the retinohypothalamic tract [15]. Peripheral clocks respond to other signals such as feeding time (the primary signal to liver circadian clocks [16]), the sleep–awake cycle, exercise, and body temperature [11].

**Figure 1:**
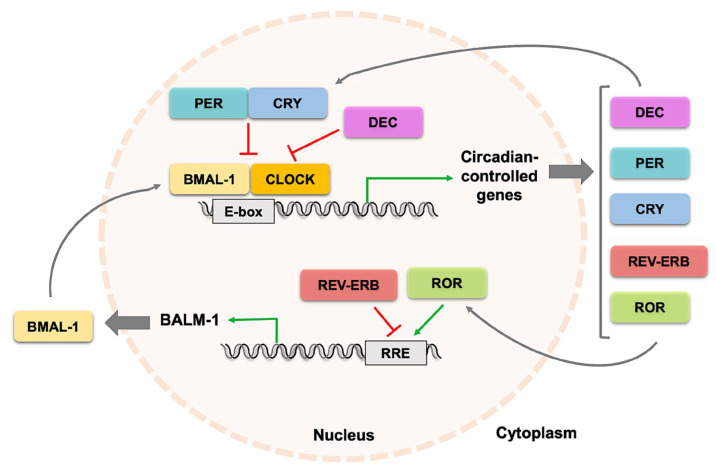
The molecular regulation of circadian clocks. The transcription factors BMAL-1 and CLOCK heterodimerize and bind to E-box elements promoting the transcription of circadian-controlled genes such as PER, CRY, DEC, REV-ERB, and ROR. Feedback loops regulate the oscillatory pattern of transcription, with PER-CRY inhibiting the transcription activity of BMAL-1-CLOCK and REV-ERB inhibiting while ROR is promoting BMAL-1 transcription. DEC competitively inhibits BMAL-CLOCK binding to the E-box element. BMAL-1, brain and muscle ARNT-like-1; CLOCK, circadian locomotor output cycle kaput; PER, period; CYR, cryptochrome; DEC, differentiation embryo chondrocytes; RORs, retinoic acid-related orphan nuclear receptors.

At the molecular level, the oscillatory rhythm is accomplished through intricate negative and positive feedback loops of gene transcription, with several autonomous feedback loops [13] (Figure 1).

The central master clock genes are the transcription factors circadian locomotor output cycle kaput (CLOCK) and brain and muscle ARNT-like 1 (BMAL-1) [17]. CLOCK and BMAL-1 heterodimers translocate into the nucleus where they bind to a specific DNA-binding sequence, E-box, in the promoters of over 300 target genes, prompting their expression [18]. Among the CLOCK-BMAL-1 target genes are the gene families period (PER-1, -2, and -3) [19] and cryptochrome (CRY-1 and -2) [20]. PER and CRY heterodimerize and function as transcription repressors, which are able to downregulate their own expression by inhibiting CLOCK-BMAL-1, which constitutes the first autoregulatory negative feedback loop [13]. The levels of PER and CRY proteins in the cytoplasm are regulated by their phosphorylation through the proteins casein kinase-1 (CK-1) [21] and F-box-LRR-repeat protein-3 (FBXL3) [22], respectively, which target them to degradation by the proteasome complex [15]. However, if PER phosphorylation occurs after PER binding to CRY, it promotes their entry into the nucleus [23], allowing the complex to inhibit CLOCK-BMAL-1.

The second autoregulatory feedback loop derives from the CLOCK-BMAL-1-induced expression of the transcription promoters retinoic acid-related orphan nuclear receptors (ROR-α, -β, -γ) and the transcription repressors REV-ERB-α, -β. RORs and REV-ERBs compete to bind retinoic acid-related orphan receptor response elements (ROREs) present in the *Bmal1* promoter, modulating BMAL-1 expression [24].

A third autoregulatory feedback loop consists in the CLOCK-BMAL-1-induced expression of the basic helix–loop–helix proteins differentiated embryo chondrocytes (DEC-1, -2), which compete with CLOCK-BMAL-1 binding to E-box, hence suppressing all their target genes’ expression [25].

Additionally, each component of the circadian clock is the target of posttranslational modifications that regulate its stability and function, such as phosphorylation, acetylation, SUMOylation, and ubiquitylation [5].

In the liver, around 40% of the transcriptome presents circadian oscillations. Posttranslational modifications also add to the circadian oscillations in hepatic protein abundance [26]. Most liver functions are under circadian rhythmicity, namely the energy homeostasis regulating carbohydrates, lipids, amino acid metabolism, protein synthesis, bile acid metabolism, detoxification, autophagy, and ER stress [5,14]. Indeed, the circadian rhythms’ extensive regulation of the liver function and of the mechanisms to cope with cellular stress justifies that a circadian rhythm dysfunction is associated with the development of MASLD and its progression to steatohepatitis, cirrhosis, and hepatocellular carcinoma [6]. Accordingly, animal models with genetically modified mice deficient in circadian clock genes (*Clock*, *Per-1/2*) develop obesity, insulin resistance, metabolic syndrome, hepatic steatosis, and fibrosing steatohepatitis, on a regular diet [27,28,29,30,31,32]. Furthermore, mice lacking *Per-1*/*2*, *Cry-1/2*, or deficient in *Bmal-1* are prone to radiation-induced tumors, including hepatocellular carcinoma [33,34]. Furthermore, *Clock* knockout in the ApoE deficient mice or in the high-fat diet MASLD models, accelerates fibrosis progression [32], cirrhosis, and hepatocellular carcinoma development [31]. Studies on human hepatocellular carcinoma showed a lower expression of clock genes in the malignant tissue as compared to non-tumoral tissue, which coupled with disturbances in the cell cycle and correlated with tumor size [35].

## 3. Disrupted Eating Patterns Are Associated with the Metabolic Syndrome, MASLD, and Hepatocellular Carcinoma

The famous quote from the XII century medieval philosopher Maimonides (also known as Rambam) “eat like a king in the morning, a prince at noon, and a peasant at dinner” summarizes the concept of chrononutrition, in which the timing of eating should be aligned with our circadian rhythms [36].

Energy homeostasis presents a circadian variation, so as to adjust to different energy requirements and food availability throughout the day. Homeostasis depends on the synchronization between the brain’s central clock which controls feeding–fasting cycles, and the peripheral clocks (such as the liver, pancreas, and skeletal muscle), which regulate metabolism to maintain normoglycemia throughout the circadian cycle [37].

During the day, food availability goes hand in hand with energy storage, with nutrient uptake (for example, BMAL-1 promotes the expression of GLUT-2 glucose transporter [38]) and the synthesis of hepatic glycogen, triglycerides, and proteins. Conversely, at night (or in situations that increase demands, such as exercise), energy derives mainly from fueling the reservoirs and gluconeogenesis [6]. Indeed, during sleeping at night, we must adapt to an extended period of total fasting [39].

Melatonin’s secretion/levels present a circadian pattern, which is regulated by the central clock in response to the circadian phase and light exposure. Melatonin levels start to increase hours before sleep, reach maximum levels during sleep, and thereafter progressively decline in the first few hours after the habitual wake time, sustaining nadir levels during the day [40]. Melatonin impairs glucose homeostasis by suppressing insulin release and insulin sensitivity [40].

Glucose tolerance also presents a 24 h cycle, which is inverse to the melatonin levels, with a lower glucose tolerance, and higher insulin response to meals, in the afternoon and night compared with the morning [39]. Interestingly, this effect is not reproduced by similar diurnal fasting in the absence of physical activity, when glucose tolerance persists, leading to a decrease in glucose levels, in clear contrast with the stable glucose levels during night sleep fasting, hence the French proverb “*qui dort, dine*”, that is, “sleeping is dinning” [39]. The circadian increase in glucose intolerance and insulin resistance at night results from sleep-dependent and independent mechanisms. Indeed, the different sleep stages present different muscle tone and uptake as well as different glucose utilization by the brain (which, for example, is higher in the REM stage compared to non-REM sleep) [41].

The melatonin oscillations have a profound impact on chrononutrition. During the day, when melatonin is low, eating is synchronized with a high glucose tolerance. At night, high melatonin levels and glucose intolerance may help maintain normoglycemia. As such, eating at night is in misalignment with higher glucose intolerance and may promote diabetogenesis [40].

The circadian oscillations displayed in insulin sensitivity and glucose tolerance may also be related to counterregulatory hormones with circadian regulation such as glucocorticoids and catecholamines.

Glucocorticoids counteract insulin and promote gluconeogenesis while inhibiting tissue glucose uptake [39]. Cortisol levels present striking circadian oscillations, with a morning maximum, decreasing during the afternoon and evening, and with a nadir around midnight [39]. The apparent contradiction with the highest glucose tolerance coinciding with the cortisol peak can be explained by the 6 to 15 h delay in the effect of glucocorticoids on insulin sensitivity [42]. Furthermore, the clock protein CRY-1 represses the glucocorticoid receptor through direct interaction, decreasing cellular response to glucocorticoids [43]. Interestingly, CRY-1 levels are higher during the night–day transition, decreasing gluconeogenesis [44].

Epinephrine also stimulates gluconeogenesis through cAMP-mediated phosphorylation of cAMP-response element binding protein (CREB) [44]. Epinephrine presents a circadian rhythm with trough levels at 3:00H AM [45].

Hepatic circadian regulation also has major roles in lipids and bile acid metabolism. Indeed, the expression of the major regulators of lipid homeostasis sterol regulatory element binding proteins (SREBPs) and peroxisome proliferator-activated receptors (PPARs) present rhythmic oscillations, which are regulated by clock genes [46,47,48,49]. Accordingly, key enzymes in lipids and bile acid metabolism are under the control of clock genes: acetyl CoA carboxylase (ACC) for lipolysis [50], hydroxymethylglutaryl-CoA reductase (HMGCR) for cholesterol metabolism [51], and cholesterol 7-α hydroxylase (CYP7A1) for bile acid synthesis [52].

### 3.1. Preclinical Evidence of Chrononutrition

Animal studies showed that not only what we eat, but also when we eat, has a profound effect on metabolism and health.

Mice are nocturnal and hence their active phase and eating time is predominantly during the evening. However, a high-fat diet tends to blunt the circadian feeding rhythms [53].

Time-restricted feeding decreases the triglycerides hepatic content in mice, even when fed a regular diet [29]. Furthermore, restricting the eating time, independently of the eating schedule being restricted to the day or the night, protected mice against obesity, insulin resistance, and MASLD induced by a high-fat diet, in parallel with improvements in the cyclic oscillations of the liver metabolome and energy homeostasis [54,55].

Lastly, feeding cycles can restore the circadian oscillations of the transcriptome in mice with a genetically disrupted clock [56].

### 3.2. Intermittent Fasting and MASLD

A 2024 meta-analysis on seven randomized controlled trials in a 2–3 month intervention with intermittent fasting on patients with MASLD, suggested a beneficial effect on body weight and abdominal adiposity, lipids and glucose profiles, and liver fat, with no effect on liver fibrosis [57]. Of note, the studies were small and highly heterogeneous regarding the type of fasting regimens (Table 1).

Intermittent fasting regimens are highly variable and can be divided into periodic fasting, in which caloric intake is limited in short periods of time, and time-restricted feeding, in which there is a time window in the day where feeding can occur [66]. Examples of periodic fasting are alternate-day fasting, which can allow up to 25% energy intake in the fasting days, and the 5:2 diet, with 2 non-consecutive days with an ad libitum diet, and a time-restricted diet for the remaining 5 days.

A time-restriction diet seems to better align with circadian oscillations; however, the feeding schedule may have an impact on its effects.

### 3.3. Eating Patterns and Obesity-Associated Diseases in Humans

Eating regular meals, as compared to engaging in erratic and irregular meal schedules, seems to prevent the metabolic syndrome, particularly to protect against insulin resistance [67]. Also, it seems to be associated with lower liver enzymes [68].

Small interventional studies suggest that eating meals earlier in the day, as compared to later, seems to promote weight loss and a healthier metabolic profile [69,70,71,72,73,74] (Table 2).

Almost one-third of the Western population does not eat breakfast [79]. Several large-scale cross-sectional and longitudinal epidemiological studies in humans showed that skipping breakfast might be associated with weight gain and obesity [80,81,82,83], even though some small cross-sectional studies failed to demonstrate it [84]. The effect ranged from a 30% to 450% increased risk in Western and Asian populations [81,82,83]. Skipping breakfast might also promote the development of the metabolic syndrome [85,86,87,88], although, again, some studies failed to demonstrate it [89,90]. Importantly, skipping breakfast has not only been associated with an increased risk of MASLD [91] but also with higher cardiovascular and cerebrovascular mortality in patients with MASLD [92]. Eating breakfast may minimize subsequent caloric intake since the ability to achieve satiety with a meal is higher at breakfast and declines throughout the day, in a macronutrient-specific fashion [93]. As such, eating carbohydrates, lipids, or proteins at breakfast is associated with lower carbohydrates, lipids, or proteins intake, respectively, during the rest of the day [93]. The satiety effect of breakfast is coupled with an increase in the satiety hormones peptide YY (PYY) and glucagon-like peptide-1 (GLP-1) [94]. Furthermore, breakfast allows for the “second meal phenomenon”, in which insulin secretion after breakfast suppresses plasma-free fatty acids, improving muscle and hepatic insulin sensitivity [95], resulting in better glycemic control after lunch [96]. Lastly, having breakfast advances the circadian phase regarding body temperature and heart rate, and promotes a shift to an early chronotype, acting as a zeitgeber endorsing synchronization with the light–dark cycle [97,98,99].

Lunchtime has also been associated with body weight. In weight loss experiments, early eaters, who had lunch before 3:00 PM, lost weight more efficiently as compared to those who had lunch after 3:00 PM, independently of energy intake, composition of the diet, and energy expenditure. Proposed mechanisms were an association between late lunch and evening meals, less energetic breakfast or the more frequent skipping of breakfast, and possible genetic polymorphisms in clock genes that could predispose to late chronotype [76,78] (Table 2).

A distribution of the energy intake with a higher fraction consumed at dinner, having a late dinner, snacking after dinner, and sleeping shortly after the last meal have also been associated with weight gain, metabolic syndrome, and MASLD [75,100,101,102,103,104,105] (Table 2).

Even though it is not consensual in epidemiological studies [81,106], eating more than four meals seems to be associated with a 15–50% increased risk of obesity [83,107,108,109] and up to a two-fold increased risk of abdominal obesity [84,108,109,110], as well as metabolic syndrome [102]. A 2023 meta-analysis of six randomized controlled trials found a trend between eating at least four meals per day and a higher body mass index (BMI) and fat mass [111]. Of note, epidemiological studies found conflicting results between eating three or fewer meals and BMI [112,113]. Lastly, eating more than three meals also seems to be associated with a 20% increased risk of MASLD (assessed by ultrasonography), with a dose-response effect, in a large-scale cross-sectional Japanese study [114].

Fast eaters, that is, those who eat a meal in less than 5–15 min, also seem to be at a higher risk of excess weight, obesity, and abdominal obesity, independently of physical activity and energy intake [115,116,117,118]. It also seems to be associated with metabolic syndrome [89]. Accordingly, fast eating seems to increase the risk of MASLD by 20% [104,119,120,121,122,123]. Fast eating abrogates the PYY and GLP-1 response to eating, failing to achieve its beneficial effects such as satiety [124].

In conclusion, the daily energy intake should decrease throughout the day, distributed in the conventional three-meal pattern, always favoring breakfast, and a light early dinner [29].

### 3.4. Intestinal Microbiota and Chrononutrition

Both mice and humans present a circadian cyclic fluctuation in the composition of the intestinal microbiota (in around 15% of the operational taxonomic units) and function, as reflected by fluctuations in one-fourth of the microbial gene expression (microbiome) [125,126,127]. The diurnal oscillations in the intestinal microbiota are modulated by feeding rhythms. The manipulation of the feeding time schedule not only shifted the kinetics of bacterial abundance but also dramatically changed the relative abundance of some bacteria, hence inducing dysbiosis [128].

In mice deficient in clock genes *Per1/2*, the circadian fluctuations of the intestinal microbiota were abrogated, but they could be restored by timed feeding. Furthermore, a high-fat diet and chronic jet lag also abrogated the rhythmicity in the microbiota composition, favoring a more obesogenic phenotype, namely with a higher Firmicutes/Bacteroidetes ratio. Alongside this, both a high-fat diet and chronic jet lag induced rapid weight gain and insulin resistance independently of the food intake. Lastly, the transfer of fecal microbiota from humans submitted to flight-induced jet lag into germ-free mice resulted in mice weight gain and in the development of insulin resistance [126].

In humans, small trials with intermittent fasting/time-restricted feeding and observations in Ramadan also showed modulation of the intestinal microbiota, toward less obesogenic (with an increase in Firmicutes) and anti-inflammatory (for example, with an increase in *Faecalibacterium*, *Butyricoccus*, and *Prevotella*), despite high heterogeneity of the results, with different bacteria popping out in those trials [129,130,131,132,133,134,135,136].

## 4. Misalignment with Light–Dark Cycles Induces the Metabolic Syndrome, MASLD, and Hepatocellular Carcinoma

The famous quote from Aristotle, in the IV BC century “it is good to rise before dawn, for such habits contribute to health, wealth and wisdom”, translates the benefits of aligning the sleep–awake cycles with our central circadian clock and light–dark natural cycles.

Circadian misalignment can be induced by exposure to artificial light during the night, rotating night shift work, and irregular sleep–awake cycles [137].

### 4.1. Artificial Light at Night (ALAN)

In mice, artificial light at night (ALAN) increases BMI and decreases glucose tolerance, in parallel with a shift in the eating time for the light phase, independently of an increase in calorie intake or physical activity [138]. ALAN exposure disrupted the core circadian rhythms in the central clock changing the mice’s feeding behaviors, and in the peripheral clocks, particularly the liver and adipose tissue, resulting in weight gain despite no change in total daily food intake [139,140]. Blue light (for example, cold-white LEDs), mainly in the 480 nm range, is particularly prone to disrupt the circadian central clock by influencing the sensor iPRGCs [141].

ALAN can result from both indoor and outdoor sources (such as streetlights, billboards, light from commercial buildings, and houses) [142]. Indoor exposure sources are lamps and devices. Importantly, 90% of the Western population use technological devices before sleep, mostly television, phones, and tablets [143]. Up to 25% of the global area of land is under light-polluted skies during the night, and 80% of the world’s population is exposed to ALAN [144].

Cross-sectional studies have suggested an association between indoor exposure to ALAN and an over 50% increased risk of obesity and abdominal obesity, dyslipidemia, and other components of the metabolic syndrome [145,146]. Insulin resistance and metabolic syndrome have been linked not only to ALAN before sleep but also to light exposure during sleep, which was coupled with an increased sympathetic tonus and altered pattern of sleep characterized by less slow wave and rapid eye movement during sleep [147,148,149]. Large-scale epidemiological studies suggest that compared with dark nights, exposure to ALAN is associated with an up to 50% increased dose-dependent risk of type 2 diabetes mellitus, independently of sleep duration, physical activity, baseline cardiometabolic health, and genetic risk [150,151,152]. Brighter night light and darker days have also been associated with a 15–20% increase in all-cause mortality, which was not modulated by sleep duration [153].

Exposure to outdoor ALAN also imposed a dose-dependent increased risk of type 2 diabetes mellitus (up to 28% increased risk), obesity (exposure associated with around 1 kg/m^2^ higher BMI), and the metabolic syndrome [154,155,156].

In addition to the direct disruptive effect on the circadian clocks of ALAN exposure, subjects reporting extensive use of devices with screens, tend to engage in less healthy diets, eating in front of the television, and higher consumption of fast food, which further increases the risk of obesity and metabolic dysfunction [157].

A simple preventive measurement to decrease the risk of metabolic dysfunction and diabetes would be to turn off the lights at night and to prefer dim and “warm” light that showed a less pronounced impact on the circadian clocks [150].

### 4.2. Rotating Night Shift Work and Long Working Hours

Submitting mice to chronic jet lag induces the spontaneous development of MASLD, steatohepatitis, fibrosis, and hepatocellular carcinoma [34,158]. Chronic jet lag induced a profound shift in the liver metabolism toward glycolysis, oxidative stress, suppression of FXR, and cholestasis. Both cholestasis and increased sympathetic tonus during sleep drove constitutive androstane receptor (CAR) overexpression, which induced hepatocarcinogenesis [34]. Chronic jet lag also heightened the hepatocarcinogenic potential of the chronic diethylnitrosamine model, which could be counterbalanced by time-restrictive diets [159].

After transmeridian travel crossing eight time zones, humans require 8 days to resynchronize the circadian clocks when traveling from west to east and 4 days from east to west [160]. A weekly 8 h night shift work schedule resembles chronic jet lag [161]. In most industrialized countries, 15–20% of workers engage in night work [162].

Small interventional studies in healthy subjects who were submitted to short-term night shift work showed an increase in glucose intolerance and insulin resistance, independently of the amount of sleep, diet, or physical activity [163,164]. Importantly, peripheral clocks such as the muscle did not align with the new behavioral cycle [163,164]. Working at night shifts is associated with eating during the evening, when insulin sensitivity is low, as dictated by the natural circadian oscillations in glucose metabolism [165].

Several meta-analyses showed that night shift workers have up to a 25% increased risk of overweight/obesity, 35% of abdominal obesity [166], over 50% of metabolic syndrome [167], and 15% of type 2 diabetes mellitus [168]. This increased risk of obesity and obesity-associated conditions presented a dose-response regarding the frequency of night shifts and the lifetime duration of night shift work [169,170,171]. Diabetic patients on night shifts present worse glycemic control, independently of body weight or caloric intake [172].

Several large-scale epidemiological studies from the USA or Asia evaluated the association between night shift work and MASLD and found an increased risk of around 25% [173,174,175,176,177,178]. Those associations were higher in subjects with normal weight compared to obese subjects [179], in younger subjects, and in women [176]. The association between night shifts and MASLD was not modified by the genetic risk of MASLD [175] and showed a dose-response for the length of night shifts (in hours), number of night shifts, and lifetime duration of night shift work [173] (Table 3).

Night shift work has also been associated, in large-scale epidemiological studies, with all-cause mortality and cardiovascular, respiratory, and digestive mortality. Subjects who engaged in night shift work for over 20 years presented a 50% increase in all-cause mortality and a two-fold increase in cardiovascular mortality [181].

The effects of night shift work could be mitigated by resynchronization of circadian clocks through timed meals. Indeed, in a randomized controlled trial in firefighters who work 24 h shifts, a time-restricted diet improved the lipids and glucose profile, as well as blood pressure in subjects with pre-existing high cardiometabolic risk, independently of caloric intake or weight loss [182].

Working long hours, that is, more than 55 h per week, seems to be associated with an increased risk of obesity [183], metabolic syndrome, type 2 diabetes mellitus [184], increased levels of aminotransferases [185], and a 25% increased risk of MASLD [184,186]. It has also been associated with cardiovascular diseases [187].

Possible explanations for the deleterious effect of working long hours could be its association with insufficient rest time and sleep quality [188], engaging in an unhealthy diet [189,190,191], and unavailability to practice exercise [192,193,194] in overworking subjects. Furthermore, long working hours have been linked to other unhealthy habits such as smoking and drinking alcohol [195].

### 4.3. Extreme Chronotype

There are two types of extreme chronotypes: (1) early larks or morning types that wake up and sleep early and, (2) night owls or evening types that wake up and sleep late.

A 2023 systematic review of seven studies showed that evening chronotype individuals are more likely to present insulin resistance and higher plasma levels of ghrelin (the “hunger hormone”), with a trend to a higher BMI [196]. In accordance, cross-sectional epidemiological studies suggested that evening chronotype/late sleepers endure a higher risk of type 2 diabetes mellitus and metabolic syndrome [197,198].

Large-scale cross-sectional Chinese studies in diabetics/prediabetics and in the general population showed a dose-dependent increase in the incidence of MASLD in those who engaged in late bedtime, after adjusting for sleep duration. They showed a 30% increase in the prevalence of MASLD per hour of late bedtime with a 50% increase when bedtime was 08:00–10:00 H PM and a two-fold increase when after 10:00 PM, as compared to before 08:00 PM [199,200]. A study taking advantage of the US NHANES confirmed an association between late sleepers, that is, those who sleep after midnight, and hepatic steatosis and fibrosis [201].

One possible explanation could be a sleep debt in late sleepers, which is associated with a decreased carbohydrate tolerance and increased sympathetic nervous system tonus [202], as well as increased caloric intake as there is more time and opportunities to eat and greater sensitivity to food reward [203]. Late sleeping results in higher exposure to ALAN. Furthermore, late sleepers more frequently engage in sedentary activities such as watching television and using electronic devices [204], and unhealthy eating habits such as more snacking, irregular meals, and drinking soft beverages [205,206].

An environmental equivalent to an evening chronotype is the geographical exposure to a later sunset. Indeed, subjects living in the Western region of a specific time zone are exposed to less light early in the day and more light later in the day, as compared to subjects living in the Eastern region of the same time zone, which promotes a shift phase and misalignment in the circadian clock. A large epidemiological study suggested that a 5-degree increase in longitude toward the west within a time zone is associated with a 7% increased risk of hepatocellular carcinoma development [207].

### 4.4. Irregular Sleep–Awake Cycles

A 2023 meta-analysis suggested that sleeping for short hours (less than 5–6 h per day) is associated with a 15% increased risk of MASLD [208], even though previous meta-analyses failed to demonstrate this association [209,210]. A large study taking advantage of the NHANES 2017–2000 pointed out the inflection point of 7.5 h per day [211]. Furthermore, bedtime sleep after midnight was associated with a 2.5-fold increased risk of significant fibrosis in patients with MASLD [211].

Social jet lag refers to short sleep duration during the week, which is compensated with longer hours of sleep during the weekends and free days [137]. The longer the gap of hours in the sleep duration between working days and the weekend, the higher the potential effects on health, coupled with a lower quality diet, obesity and central obesity, metabolic syndrome, and type 2 diabetes mellitus, particularly when over 2 h [212,213,214,215]. A social jet lag of at least 2 h was reported in 50% of workers and students [137].

Regarding day napping, a 2024 meta-analysis suggested that long naps (that is, longer than 30 min) during the day are associated with a 20% increased risk of MASLD [216]. A Mendelian randomization analysis of GWAS also found a positive correlation between day napping and hepatocellular carcinoma [217]. The possible mechanisms of the deleterious effect of day napping could be a tendency to night late sleeping and short sleep duration, and an increase in cortisol and sympathetic tonus [218,219].

A study taking advantage of the National Health Insurance Research Database from Taiwan found a 44% increased standardized incidence of hepatocellular carcinoma in patients with sleep disorders (after the exclusion of sleep apnea) [220]. Lastly, a Mendelian randomization analysis of GWAS found that sleep duration negatively correlated and insomnia positively correlated with the risk of hepatocellular carcinoma [217].

## 5. Conclusions

Circadian rhythms have a profound impact on the regulation of metabolism, particularly hepatic metabolism. Liver clocks are subsidiary to the central clock in the hypothalamus that synchronizes predominantly with the natural light–dark cycle. Liver clocks also oscillate autonomously in response to feeding–fasting cycles. Circadian misalignment of the liver clocks promotes metabolic dysfunction, MASLD, and hepatocarcinogenesis.

From the realization of the circadian modulation in the pathogenesis of MASLD, a new approach is gaining enthusiasm: chrononutrition, the concept that not only what we eat, but when we eat can modulate health and disease. Indeed, time-restricted diets could be of help, depending on the feeding time schedule. Caloric ingestion should start with breakfast, and decrease throughout the day, with an early last meal, and no dietary guilty pleasures after dinner. The classical three meals distribution seems suitable, and additional meals or snacks should be avoided.

Another concept that should be explored more is chronopharmacology since most targets for the treatment of MASLD (for example, thyroid hormone receptor, GLP-1, and FGF-21) are under circadian oscillation.

Lastly, circadian sleep–awake cycle misalignment, such as night shift schedules, seems to strongly negatively affect metabolic health and promote MASLD development. Resynchronization through time-restricted diets may be a simple, effective strategy to mitigate those deleterious effects.

## Figures and Tables

**Table 1:** Summary of randomized clinical trials on the effects of intermittent fasting in MASLD.

Study	Country	N	Population	Intervention	Outcomes
Johari, 2019 [58]	Malaysia	33	Adults with MASLD and ↑ aminotransferases	2-month intervention Modified ADF	IF was associated with 30%↓ in ALT, 25%↓ in steatosis, and 15%↓ in fibrosis by SWE
Cai, 2019 [59]	China	271	Adults with MASLD and ≥F3 by Fibroscan^©^ (≥9.6 kPa)	3-month intervention ADF vs. TRE vs. control	↓ 5% body weight in ADF, 4% in TRE, and 2.5% in controls. No effect on fibrosis by Fibroscan^©^
Homer, 2021 [60]	Sweden	74	Adults with MASLD by imaging or Fibroscan^©^ (CAP ≥ 280 dB/m)	3-month intervention 5:2 IF diet or LCHF diet vs. control	IF and LCHF with 6%↓ and 7%↓ in IHTG (vs. 3.6%↓ in controls) IF with −1.5 kPa↓ in fibrosis by Fibroscan^©^
Kord Varkaneh, 2022 [61]	Iran	44	Adults with MASLD by Fibroscan^©^ (CAP ≥ 260 dB/m)	3-month intervention 5:2 IF diet	IF was associated with 33%↓ in ALT, 7%↓ in steatosis, and 20%↓ in fibrosis by Fibroscan^©^
Ezpeleta, 2023 [62]	USA	80	Adults with obesity and MASLD (↑ IHTG by MRI)	3-month intervention ADF and/or moderately intensive aerobic exercise (5 × 60 min/week)	ADF ± exercise was associated with 5% ↓ in IHTG compared with control (0.2%↓)
Sun, 2024 [63]	China	60	Adults with MASLD (↑ IHTG by MRI)	3-month intervention 5:2 IF or CRD	Trend toward higher ↓ in IHTG in IF (25% vs. 15%) Higher ↓ in HbA1c, ≅ ↓ in weight
Lee, 2024 [64]	South Korea	72	Adults with obesity and MASLD (↑ IHTG by MRI)	3-month intervention 5:2 IF diet	IF was associated with more often ↓ in IHTG ≥33% (72% vs. 44%) and ≅ ↓ in weight (5%)
Wang, 2024 [65]	China	60	Adults with MASLD (imaging or biopsy)	3-month intervention 5:2 IF or CRD	5:2 IF associated with higher ↓ in steatosis (24% vs. 16%) and fibrosis (28% vs. 17%) by Fibroscan^©^, compared with CRD

ADF, alternate-day fasting; CAP, controlled attenuation parameter; CRD, calorie-restricted diet; IF, intermittent fasting; IHTG, intrahepatic triglycerides content; LCHF, low carb high fat; MASLD, metabolic dysfunction-associated steatotic liver disease; MRI; magnetic resonance imaging; SWE, stress wave elastography; TRE, time-restricted diet; ↑, elevation; ↓, decrease.

**Table 2:** Summary of interventional studies on chrononutrition and metabolic dysfunction or MASLD.

Study	Country	N	Population	Intervention	Outcomes
LeCheminant, 2013 [69]	USA	27	Healthy men (age 21 ± 2 years)	2-week intervention NER (no E intake 7 PM to 6 AM) vs. control diet	NER associated with ↓ E intake (10,125 vs. 11,146 kJ) and ↓ weight (−0.4 vs. +0.6 kg)
Hibi, 2013 [71]	Japan	11	Healthy women (age 23 ± 1 years)	13-day intervention Daytime (10 AM) vs. nighttime (23 PM) snacking (200 kcal)	Nighttime snacking ↑ total/LDL- cholesterol and ↓ fat oxidation
Yoshizaki, 2013 [73]	Japan	14	Healthy men (age 21 ± 1 years)	2-week intervention Early (8 AM, 1 PM, 6 PM) vs. delayed (1 PM, 6 PM, 23 PM) mealtime	Early mealtime ↓ triglycerides and total/LDL-cholesterol
Jakubowicz, 2013 [75]	Israel	93	Overweight and obese women (age 45 ± 1 years)	2-week intervention 50% caloric intake at breakfast vs. at dinner	High calories at breakfast ↓ IR more than at dinner and ↓ triglycerides 33% (vs. ↑ 14%)
Garaulet, 2013 [76]	Spain	420	Overweight and obese subjects (age 42 ± 11 years)	20-week weight loss trial Early vs. late eaters (lunch before or after 3 PM)	Early eaters with higher weight loss (11 vs. 9%) despite ≅ E intake
Koopman, 2014 [77]	The Netherlands	36	Healthy men (age 23 ± 3 years)	6-week intervention 40% hypercaloric diet ↑ meal size or frequency (3 vs. 5–6 meals/day)	↑ frequency, but not meal size, ↑ IHTG (45 for HFHS and 110% for HS), ↑ abdominal fat, and ↓ hepatic insulin sensitivity
Bandin, 2015 [78]	Spain	32	Healthy women (age 24 ± 4 years)	2-week intervention Early (1 PM) vs. late lunch (4 PM)	Late lunch is associated with ↓ resting E expenditure and ↓ glucose tolerance
Singh, 2020 [72]	India	33	Healthy adults (age 27 ± 10 years)	4-week intervention Morning (8–9.3 AM) vs. evening (8–9:3 PM) eating	Evening eating ↑ weight (0.5 kg), BMI (0.3 kg/m^2^), WC (1.13 cm), SBP, and glycated Hb (0.28%)
Rangaraj, 2020 [74]	USA	44	Healthy adults (age 21–50 years)	3-day food diary	Higher proportion of E intake in the morning was associated with ↑ insulin sensitivity
Allison, 2021 [70]	USA	12	Healthy adults (age 26 ± 3 years)	2-week intervention Daytime (8 AM to 19 PM) vs. delayed (12 PM to 23 PM) eating	Weight, IR, trunk-to-leg fat ratio, resting E expenditure, and total cholesterol ↓ in daytime vs. delayed schedule

BMI, body mass index; E, energy; Hb, hemoglobin; HFHS, high fat high sugar; HS, high sugar; IHTG, intrahepatic triglycerides content; IR, insulin resistance; NER, night eating restriction; SBP, systolic blood pressure; WC, waist circumference; ↑, increase; ↓, decrease.

**Table 3:** Summary of epidemiological studies on the effect of night shift work in the risk of MASLD development.

Study	Country	N	Design	Major Findings
Balakrishnan, 2017 [179]	USA	8159	NHANES-based cross-sectional study (cycles 2005–2010). MASLD by ↑ aminotransferases.	NSW was not an independent risk factor for MASLD (even though MASLD was more frequent among participants who engaged in NSW: 17% vs. 15.5%). In the subgroup with normal BMI, NSW was associated with a 66% ↑ risk of MASLD.
Zhang, 2020 [180]	China	6881	Cross-sectional study in steelworkers with different exposure to NSW. MASLD by US.	Steelworkers currently on NSW presented a 23% ↑ risk of MASLD vs. those who never engaged in NSW; >7 NSW/month associated with a 24% and >8 h/night 27% ↑ risk of MASLD.
Kim, 2022 [178]	South Korea	2511	Cross-sectional study in male steelworkers with different exposure to NSW. MASLD by US.	NSW associated with a 45% ↑ risk of moderate–severe MASLD, particular higher if NSW for > 20 years (↑ 228%), and when eating during NSW (↑ 158%).
Huang, 2023 [175]	UK	281,280	Prospective analysis of UK Biobank participants, followed for 12 years. Medical diagnosis of MASLD.	Some NSW or usual/ permanent NSW associated with a 12% and 27% ↑ risk of MASLD, respectively. Duration and frequency of NSW, number of consecutive nights, and number of hours/NSW positively associated with incident MASLD. Genetic predisposition to MASLD did not modify the association between NSW and incident MASLD.
Xu, 2023 [177]	China	14,112	A 4-year longitudinal cohort study on railworkers. MASLD by US.	Compared with seldom NSW, occasional NSW associated with a 7% ↑ risk of MASLD and frequent NSW with a 18% ↑.
Lee, 2024 [176]	South Korea	45,149	Longitudinal analysis of participants of the Kangbuk Samsung Health Study. MASLD by US.	Participants in their 20s, presented an association between NSW and a 24% ↑ risk of incident MASLD.
Maidstone, 2024 [174]	UK	282,303	Cross-sectional analysis of UK Biobank participants. MASLD by ↑ IHTG.	Irregular NSW associated with a 29% ↑ risk of MASLD and permanent NSW with 8% ↑. The effect was partially mediated by the ↑ in BMI.

MASLD, metabolic dysfunction-associated steatotic liver disease; NSW, night shift work; US, ultrasound; ↑, increased.

## Abbreviations

ACC: acetyl CoA carboxylase; ALAN, artificial light at night; BMAL-1, brain and muscle ARNT-like 1; BMI, body mass index; CAR, constitutive androstane receptor; CLOCK, circadian locomotor output cycle kaput; CK1, casein kinase-1; CRY, cryptochrome; CYP7A1, cholesterol 7-α hydroxylase; DEC, basic helix–loop–helix proteins differentiated embryo; FBXL3, F-box-LRR-repeat protein 3; GLP-1, glucagon-like peptide 1; HMGCR, hydroxymethylglutaryl-CoA reductase; ipRGC, photoreceptive retinal ganglion cell; MASLD, metabolic dysfunction-associated steatotic liver disease; PPARs, peroxisome proliferator-activated receptors; PER, period; PYY, peptide YY; RORs, retinoic acid-related orphan nuclear receptors; RORE, retinoic acid-related orphan receptor response element; SREBPs, sterol regulatory element binding proteins.

## References

[1] Lingvay I., Cohen R.V., Roux C.W.L., Sumithran P. (2024). Obesity in adults. Lancet.

[2] Neeland I.J., Lim S., Tchernof A., Gastaldelli A., Rangaswami J., Ndumele C.E., Powell-Wiley T.M., Despres J.P. (2024). Metabolic syndrome. Nat. Rev. Dis. Primers.

[3] Younossi Z.M., Golabi P., Paik J.M., Henry A., Van Dongen C., Henry L. (2023). The global epidemiology of nonalcoholic fatty liver disease (nafld) and nonalcoholic steatohepatitis (nash): A systematic review. Hepatology.

[4] Eslam M., George J. (2020). Genetic contributions to nafld: Leveraging shared genetics to uncover systems biology. Nat. Rev. Gastroenterol. Hepatol..

[5] Saran A.R., Dave S., Zarrinpar A. (2020). Circadian rhythms in the pathogenesis and treatment of fatty liver disease. Gastroenterology.

[6] Bolshette N., Ibrahim H., Reinke H., Asher G. (2023). Circadian regulation of liver function: From molecular mechanisms to disease pathophysiology. Nat. Rev. Gastroenterol. Hepatol..

[7] Halberg F., Cornelissen G., Katinas G., Syutkina E.V., Sothern R.B., Zaslavskaya R., Halberg F., Watanabe Y., Schwartzkopff O., Otsuka K. (2003). Transdisciplinary unifying implications of circadian findings in the 1950s. J. Circadian Rhythms.

[8] Chambers L., Seidler K., Barrow M. (2023). Circadian misalignment in obesity: The role for time-restricted feeding. Clin. Nutr. ESPEN.

[9] Javeed N., Matveyenko A.V. (2018). Circadian etiology of type 2 diabetes mellitus. Physiology.

[10] Zimmet P., Alberti K., Stern N., Bilu C., El-Osta A., Einat H., Kronfeld-Schor N. (2019). The circadian syndrome: Is the metabolic syndrome and much more!. J. Intern. Med..

[11] Mukherji A., Bailey S.M., Staels B., Baumert T.F. (2019). The circadian clock and liver function in health and disease. J. Hepatol..

[12] Rajan P.K., Udoh U.S., Finley R., Pierre S.V., Sanabria J. (2024). The biological clock of liver metabolism in metabolic dysfunction-associated steatohepatitis progression to hepatocellular carcinoma. Biomedicines.

[13] Ko C.H., Takahashi J.S. (2006). Molecular components of the mammalian circadian clock. Hum. Mol. Genet..

[14] Reinke H., Asher G. (2016). Circadian clock control of liver metabolic functions. Gastroenterology.

[15] Fagiani F., Di Marino D., Romagnoli A., Travelli C., Voltan D., Di Cesare Mannelli L., Racchi M., Govoni S., Lanni C. (2022). Molecular regulations of circadian rhythm and implications for physiology and diseases. Signal Transduct. Target. Ther..

[16] Stokkan K.A., Yamazaki S., Tei H., Sakaki Y., Menaker M. (2001). Entrainment of the circadian clock in the liver by feeding. Science.

[17] Gekakis N., Staknis D., Nguyen H.B., Davis F.C., Wilsbacher L.D., King D.P., Takahashi J.S., Weitz C.J. (1998). Role of the clock protein in the mammalian circadian mechanism. Science.

[18] Yan J., Wang H., Liu Y., Shao C. (2008). Analysis of gene regulatory networks in the mammalian circadian rhythm. PLoS Comput. Biol..

[19] Zheng B., Albrecht U., Kaasik K., Sage M., Lu W., Vaishnav S., Li Q., Sun Z.S., Eichele G., Bradley A. (2001). Nonredundant roles of the mper1 and mper2 genes in the mammalian circadian clock. Cell.

[20] Kume K., Zylka M.J., Sriram S., Shearman L.P., Weaver D.R., Jin X., Maywood E.S., Hastings M.H., Reppert S.M. (1999). Mcry1 and mcry2 are essential components of the negative limb of the circadian clock feedback loop. Cell.

[21] Knippschild U., Gocht A., Wolff S., Huber N., Lohler J., Stoter M. (2005). The casein kinase 1 family: Participation in multiple cellular processes in eukaryotes. Cell Signal.

[22] Busino L., Bassermann F., Maiolica A., Lee C., Nolan P.M., Godinho S.I., Draetta G.F., Pagano M. (2007). Scffbxl3 controls the oscillation of the circadian clock by directing the degradation of cryptochrome proteins. Science.

[23] Vielhaber E., Eide E., Rivers A., Gao Z.H., Virshup D.M. (2000). Nuclear entry of the circadian regulator mper1 is controlled by mammalian casein kinase i epsilon. Mol. Cell Biol..

[24] Guillaumond F., Dardente H., Giguere V., Cermakian N. (2005). Differential control of bmal1 circadian transcription by rev-erb and ror nuclear receptors. J. Biol. Rhythms.

[25] Honma S., Kawamoto T., Takagi Y., Fujimoto K., Sato F., Noshiro M., Kato Y., Honma K. (2002). Dec1 and dec2 are regulators of the mammalian molecular clock. Nature.

[26] Daniels L.J., Kay D., Marjot T., Hodson L., Ray D.W. (2023). Circadian regulation of liver metabolism: Experimental approaches in human, rodent, and cellular models. Am. J. Physiol. Cell Physiol..

[27] Turek F.W., Joshu C., Kohsaka A., Lin E., Ivanova G., McDearmon E., Laposky A., Losee-Olson S., Easton A., Jensen D.R. (2005). Obesity and metabolic syndrome in circadian clock mutant mice. Science.

[28] Yang S., Liu A., Weidenhammer A., Cooksey R.C., McClain D., Kim M.K., Aguilera G., Abel E.D., Chung J.H. (2009). The role of mper2 clock gene in glucocorticoid and feeding rhythms. Endocrinology.

[29] Adamovich Y., Rousso-Noori L., Zwighaft Z., Neufeld-Cohen A., Golik M., Kraut-Cohen J., Wang M., Han X., Asher G. (2014). Circadian clocks and feeding time regulate the oscillations and levels of hepatic triglycerides. Cell Metab..

[30] Jacobi D., Liu S., Burkewitz K., Kory N., Knudsen N.H., Alexander R.K., Unluturk U., Li X., Kong X., Hyde A.L. (2015). Hepatic bmal1 regulates rhythmic mitochondrial dynamics and promotes metabolic fitness. Cell Metab..

[31] Pan X., Queiroz J., Hussain M.M. (2020). Nonalcoholic fatty liver disease in clock mutant mice. J. Clin. Investig..

[32] Yang S., Ren X., Liu J., Lei Y., Li M., Wang F., Cheng S., Ying J., Ding J., Chen X. (2024). Knockdown of the clock gene in the liver aggravates masld in mice via inhibiting lipophagy. Mol. Cell Biochem..

[33] Lee S., Donehower L.A., Herron A.J., Moore D.D., Fu L. (2010). Disrupting circadian homeostasis of sympathetic signaling promotes tumor development in mice. PLoS ONE.

[34] Kettner N.M., Voicu H., Finegold M.J., Coarfa C., Sreekumar A., Putluri N., Katchy C.A., Lee C., Moore D.D., Fu L. (2016). Circadian homeostasis of liver metabolism suppresses hepatocarcinogenesis. Cancer Cell.

[35] Lin Y.M., Chang J.H., Yeh K.T., Yang M.Y., Liu T.C., Lin S.F., Su W.W., Chang J.G. (2008). Disturbance of circadian gene expression in hepatocellular carcinoma. Mol. Carcinog..

[36] Asher G., Sassone-Corsi P. (2015). Time for food: The intimate interplay between nutrition, metabolism, and the circadian clock. Cell.

[37] Kalsbeek A., la Fleur S., Fliers E. (2014). Circadian control of glucose metabolism. Mol. Metab..

[38] Manella G., Sabath E., Aviram R., Dandavate V., Ezagouri S., Golik M., Adamovich Y., Asher G. (2021). The liver-clock coordinates rhythmicity of peripheral tissues in response to feeding. Nat. Metab..

[39] Van Cauter E., Polonsky K.S., Scheen A.J. (1997). Roles of circadian rhythmicity and sleep in human glucose regulation. Endocr. Rev..

[40] Garaulet M., Qian J., Florez J.C., Arendt J., Saxena R., Scheer F. (2020). Melatonin effects on glucose metabolism: Time to unlock the controversy. Trends Endocrinol. Metab..

[41] Van Cauter E., Blackman J.D., Roland D., Spire J.P., Refetoff S., Polonsky K.S. (1991). Modulation of glucose regulation and insulin secretion by circadian rhythmicity and sleep. J. Clin. Investig..

[42] Plat L., Byrne M.M., Sturis J., Polonsky K.S., Mockel J., Fery F., Van Cauter E. (1996). Effects of morning cortisol elevation on insulin secretion and glucose regulation in humans. Am. J. Physiol..

[43] Lamia K.A., Papp S.J., Yu R.T., Barish G.D., Uhlenhaut N.H., Jonker J.W., Downes M., Evans R.M. (2011). Cryptochromes mediate rhythmic repression of the glucocorticoid receptor. Nature.

[44] Zhang E.E., Liu Y., Dentin R., Pongsawakul P.Y., Liu A.C., Hirota T., Nusinow D.A., Sun X., Landais S., Kodama Y. (2010). Cryptochrome mediates circadian regulation of camp signaling and hepatic gluconeogenesis. Nat. Med..

[45] Linsell C.R., Lightman S.L., Mullen P.E., Brown M.J., Causon R.C. (1985). Circadian rhythms of epinephrine and norepinephrine in man. J. Clin. Endocrinol. Metab..

[46] Chaix A., Lin T., Le H.D., Chang M.W., Panda S. (2019). Time-restricted feeding prevents obesity and metabolic syndrome in mice lacking a circadian clock. Cell Metab..

[47] Le Martelot G., Claudel T., Gatfield D., Schaad O., Kornmann B., Lo Sasso G., Moschetta A., Schibler U. (2009). Rev-erbalpha participates in circadian srebp signaling and bile acid homeostasis. PLoS Biol..

[48] Zhang Y., Papazyan R., Damle M., Fang B., Jager J., Feng D., Peed L.C., Guan D., Sun Z., Lazar M.A. (2017). The hepatic circadian clock fine-tunes the lipogenic response to feeding through roralpha/gamma. Genes. Dev..

[49] Shirai H., Oishi K., Kudo T., Shibata S., Ishida N. (2007). Pparalpha is a potential therapeutic target of drugs to treat circadian rhythm sleep disorders. Biochem. Biophys. Res. Commun..

[50] Davies S.P., Carling D., Munday M.R., Hardie D.G. (1992). Diurnal rhythm of phosphorylation of rat liver acetyl-coa carboxylase by the amp-activated protein kinase, demonstrated using freeze-clamping. Effects of high fat diets. Eur. J. Biochem..

[51] Kudo T., Kawashima M., Tamagawa T., Shibata S. (2008). Clock mutation facilitates accumulation of cholesterol in the liver of mice fed a cholesterol and/or cholic acid diet. Am. J. Physiol. Endocrinol. Metab..

[52] Duez H., van der Veen J.N., Duhem C., Pourcet B., Touvier T., Fontaine C., Derudas B., Bauge E., Havinga R., Bloks V.W. (2008). Regulation of bile acid synthesis by the nuclear receptor rev-erbalpha. Gastroenterology.

[53] Kohsaka A., Laposky A.D., Ramsey K.M., Estrada C., Joshu C., Kobayashi Y., Turek F.W., Bass J. (2007). High-fat diet disrupts behavioral and molecular circadian rhythms in mice. Cell Metab..

[54] Hatori M., Vollmers C., Zarrinpar A., DiTacchio L., Bushong E.A., Gill S., Leblanc M., Chaix A., Joens M., Fitzpatrick J.A. (2012). Time-restricted feeding without reducing caloric intake prevents metabolic diseases in mice fed a high-fat diet. Cell Metab..

[55] Sherman H., Genzer Y., Cohen R., Chapnik N., Madar Z., Froy O. (2012). Timed high-fat diet resets circadian metabolism and prevents obesity. FASEB J..

[56] Vollmers C., Gill S., DiTacchio L., Pulivarthy S.R., Le H.D., Panda S. (2009). Time of feeding and the intrinsic circadian clock drive rhythms in hepatic gene expression. Proc. Natl. Acad. Sci. USA.

[57] Saleh S.A.K., Santos H.O., Gaman M.A., Cerqueira H.S., Zaher E.A., Alromaih W.R., Arafat N.S., Adi A.R., Adly H.M., Alyoubi R. (2024). Effects of intermittent fasting regimens on glycemic, hepatic, anthropometric, and clinical markers in patients with non-alcoholic fatty liver disease: Systematic review and meta-analysis of randomized controlled trials. Clin. Nutr. ESPEN.

[58] Johari M.I., Yusoff K., Haron J., Nadarajan C., Ibrahim K.N., Wong M.S., Hafidz M.I.A., Chua B.E., Hamid N., Arifin W.N. (2019). A randomised controlled trial on the effectiveness and adherence of modified alternate-day calorie restriction in improving activity of non-alcoholic fatty liver disease. Sci. Rep..

[59] Cai H., Qin Y.L., Shi Z.Y., Chen J.H., Zeng M.J., Zhou W., Chen R.Q., Chen Z.Y. (2019). Effects of alternate-day fasting on body weight and dyslipidaemia in patients with non-alcoholic fatty liver disease: A randomised controlled trial. BMC Gastroenterol..

[60] Holmer M., Lindqvist C., Petersson S., Moshtaghi-Svensson J., Tillander V., Brismar T.B., Hagstrom H., Stal P. (2021). Treatment of nafld with intermittent calorie restriction or low-carb high-fat diet—A randomised controlled trial. JHEP Rep..

[61] Kord Varkaneh H., Salehi Sahlabadi A., Gaman M.A., Rajabnia M., Sedanur Macit-Celebi M., Santos H.O., Hekmatdoost A. (2022). Effects of the 5:2 intermittent fasting diet on non-alcoholic fatty liver disease: A randomized controlled trial. Front. Nutr..

[62] Ezpeleta M., Gabel K., Cienfuegos S., Kalam F., Lin S., Pavlou V., Song Z., Haus J.M., Koppe S., Alexandria S.J. (2023). Effect of alternate day fasting combined with aerobic exercise on non-alcoholic fatty liver disease: A randomized controlled trial. Cell Metab..

[63] Sun X., Li F., Yan H., Chang X., Yao X., Yang X., Wu S., Suo Y., Zhu X., Wang C. (2024). Intermittent compared with continuous calorie restriction for treatment of metabolic dysfunction-associated steatotic liver disease: A randomized clinical trial. Am. J. Clin. Nutr..

[64] Lee H.A., Moon H., Kim Y., Lee J.K., Lee H.A., Kim H.Y. (2024). Effects of intermittent calorie restriction in nondiabetic patients with metabolic dysfunction-associated steatotic liver disease. Clin. Gastroenterol. Hepatol..

[65] Wang Y.Y., Tian F., Qian X.L., Ying H.M., Zhou Z.F. (2024). Effect of 5:2 intermittent fasting diet versus daily calorie restriction eating on metabolic-associated fatty liver disease-a randomized controlled trial. Front. Nutr..

[66] Marjot T., Tomlinson J.W., Hodson L., Ray D.W. (2023). Timing of energy intake and the therapeutic potential of intermittent fasting and time-restricted eating in nafld. Gut.

[67] Farshchi H.R., Taylor M.A., Macdonald I.A. (2005). Beneficial metabolic effects of regular meal frequency on dietary thermogenesis, insulin sensitivity, and fasting lipid profiles in healthy obese women. Am. J. Clin. Nutr..

[68] Sierra-Johnson J., Unden A.L., Linestrand M., Rosell M., Sjogren P., Kolak M., De Faire U., Fisher R.M., Hellenius M.L. (2008). Eating meals irregularly: A novel environmental risk factor for the metabolic syndrome. Obesity.

[69] LeCheminant J.D., Christenson E., Bailey B.W., Tucker L.A. (2013). Restricting night-time eating reduces daily energy intake in healthy young men: A short-term cross-over study. Br. J. Nutr..

[70] Allison K.C., Hopkins C.M., Ruggieri M., Spaeth A.M., Ahima R.S., Zhang Z., Taylor D.M., Goel N. (2021). Prolonged, controlled daytime versus delayed eating impacts weight and metabolism. Curr. Biol..

[71] Hibi M., Masumoto A., Naito Y., Kiuchi K., Yoshimoto Y., Matsumoto M., Katashima M., Oka J., Ikemoto S. (2013). Nighttime snacking reduces whole body fat oxidation and increases ldl cholesterol in healthy young women. Am. J. Physiol. Regul. Integr. Comp. Physiol..

[72] Singh R.B., Cornelissen G., Mojto V., Fatima G., Wichansawakun S., Singh M., Kartikey K., Sharma J.P., Torshin V.I., Chibisov S. (2020). Effects of circadian restricted feeding on parameters of metabolic syndrome among healthy subjects. Chronobiol. Int..

[73] Yoshizaki T., Tada Y., Hida A., Sunami A., Yokoyama Y., Yasuda J., Nakai A., Togo F., Kawano Y. (2013). Effects of feeding schedule changes on the circadian phase of the cardiac autonomic nervous system and serum lipid levels. Eur. J. Appl. Physiol..

[74] Rangaraj V.R., Siddula A., Burgess H.J., Pannain S., Knutson K.L. (2020). Association between timing of energy intake and insulin sensitivity: A cross-sectional study. Nutrients.

[75] Jakubowicz D., Barnea M., Wainstein J., Froy O. (2013). High caloric intake at breakfast vs. Dinner differentially influences weight loss of overweight and obese women. Obesity.

[76] Garaulet M., Gomez-Abellan P., Alburquerque-Bejar J.J., Lee Y.C., Ordovas J.M., Scheer F.A. (2013). Timing of food intake predicts weight loss effectiveness. Int. J. Obes..

[77] Koopman K.E., Caan M.W., Nederveen A.J., Pels A., Ackermans M.T., Fliers E., la Fleur S.E., Serlie M.J. (2014). Hypercaloric diets with increased meal frequency, but not meal size, increase intrahepatic triglycerides: A randomized controlled trial. Hepatology.

[78] Bandin C., Scheer F.A., Luque A.J., Avila-Gandia V., Zamora S., Madrid J.A., Gomez-Abellan P., Garaulet M. (2015). Meal timing affects glucose tolerance, substrate oxidation and circadian-related variables: A randomized, crossover trial. Int. J. Obes..

[79] St-Onge M.P., Ard J., Baskin M.L., Chiuve S.E., Johnson H.M., Kris-Etherton P., Varady K., On behalf of the American Heart Association Obesity Committee of the Council on Lifestyle and Cardiometabolic Health, Council on Cardiovascular Disease in the Young, Council on Clinical Cardiology (2017). Meal timing and frequency: Implications for cardiovascular disease prevention: A scientific statement from the american heart association. Circulation.

[80] Mesas A.E., Munoz-Pareja M., Lopez-Garcia E., Rodriguez-Artalejo F. (2012). Selected eating behaviours and excess body weight: A systematic review. Obes. Rev..

[81] Ma Y., Bertone E.R., Stanek E.J., Reed G.W., Hebert J.R., Cohen N.L., Merriam P.A., Ockene I.S. (2003). Association between eating patterns and obesity in a free-living us adult population. Am. J. Epidemiol..

[82] Goto M., Kiyohara K., Kawamura T. (2010). Lifestyle risk factors for overweight in Japanese male college students. Public Health Nutr..

[83] van der Heijden A.A., Hu F.B., Rimm E.B., van Dam R.M. (2007). A prospective study of breakfast consumption and weight gain among U.S. men. Obesity.

[84] Tamez M., Rodriguez-Orengo J.F., Mattei J. (2020). Higher eating frequency, but not skipping breakfast, is associated with higher odds of abdominal obesity in adults living in puerto rico. Nutr. Res..

[85] Katsuura-Kamano S., Arisawa K., Uemura H., Van Nguyen T., Takezaki T., Ibusuki R., Suzuki S., Otani T., Okada R., Kubo Y. (2021). Association of skipping breakfast and short sleep duration with the prevalence of metabolic syndrome in the general japanese population: Baseline data from the japan multi-institutional collaborative cohort study. Prev. Med. Rep..

[86] Kim H.M., Kang H.J., Lee D.H., Jeong S.M., Joh H.K. (2023). Association between breakfast frequency and metabolic syndrome among young adults in South Korea. Sci. Rep..

[87] Lujan-Barroso L., Iglesias L., Zamora-Ros R., Lasheras C., Sanchez M.J., Cabrera-Castro N., Delfrad J., Amiano P., Molina-Montes E., Colorado-Yohar S. (2023). Breakfast size and prevalence of metabolic syndrome in the european prospective investigation into cancer and nutrition (epic) spanish cohort. Nutrients.

[88] Deshmukh-Taskar P., Nicklas T.A., Radcliffe J.D., O’Neil C.E., Liu Y. (2013). The relationship of breakfast skipping and type of breakfast consumed with overweight/obesity, abdominal obesity, other cardiometabolic risk factors and the metabolic syndrome in young adults. The national health and nutrition examination survey (nhanes): 1999–2006. Public Health Nutr..

[89] Shin A., Lim S.Y., Sung J., Shin H.R., Kim J. (2009). Dietary intake, eating habits, and metabolic syndrome in Korean men. J. Am. Diet. Assoc..

[90] Jung J., Kim A.S., Ko H.J., Choi H.I., Hong H.E. (2020). Association between breakfast skipping and the metabolic syndrome: The Korea national health and nutrition examination survey, 2017. Medicina.

[91] Han A.L. (2020). Association between non-alcoholic fatty liver disease and dietary habits, stress, and health-related quality of life in Korean adults. Nutrients.

[92] Xie J., Huang H., Chen Y., Xu L., Xu C. (2022). Skipping breakfast is associated with an increased long-term cardiovascular mortality in metabolic dysfunction-associated fatty liver disease (mafld) but not mafld-free individuals. Aliment. Pharmacol. Ther..

[93] de Castro J.M. (2009). When, how much and what foods are eaten are related to total daily food intake. Br. J. Nutr..

[94] Forester S.M., Widaman A.M., Krishnan S., Witbracht M.G., Horn W.F., Laugero K.D., Keim N.L. (2018). A clear difference emerges in hormone patterns following a standard midday meal in young women who regularly eat or skip breakfast. J. Nutr..

[95] Jovanovic A., Leverton E., Solanky B., Ravikumar B., Snaar J.E., Morris P.G., Taylor R. (2009). The second-meal phenomenon is associated with enhanced muscle glycogen storage in humans. Clin. Sci..

[96] Meng H., Matthan N.R., Ausman L.M., Lichtenstein A.H. (2017). Effect of prior meal macronutrient composition on postprandial glycemic responses and glycemic index and glycemic load value determinations. Am. J. Clin. Nutr..

[97] Krauchi K., Cajochen C., Werth E., Wirz-Justice A. (2002). Alteration of internal circadian phase relationships after morning versus evening carbohydrate-rich meals in humans. J. Biol. Rhythms.

[98] Ogata H., Horie M., Kayaba M., Tanaka Y., Ando A., Park I., Zhang S., Yajima K., Shoda J.I., Omi N. (2020). Skipping breakfast for 6 days delayed the circadian rhythm of the body temperature without altering clock gene expression in human leukocytes. Nutrients.

[99] Harada T., Hirotani M., Maeda M., Nomura H., Takeuchi H. (2007). Correlation between breakfast tryptophan content and morning-evening in Japanese infants and students aged 0–15 yrs. J. Physiol. Anthropol..

[100] Hermenegildo-Lopez Y., Donat-Vargas C., Sandoval-Insausti H., Moreno-Franco B., Rodriguez-Ayala M., Rey-Garcia J., Banegas J.R., Rodriguez-Artalejo F., Guallar-Castillon P. (2021). A higher intake of energy at dinner is associated with incident metabolic syndrome: A prospective cohort study in older adults. Nutrients.

[101] Kutsuma A., Nakajima K., Suwa K. (2014). Potential association between breakfast skipping and concomitant late-night-dinner eating with metabolic syndrome and proteinuria in the Japanese population. Scientifica.

[102] Ha K., Song Y. (2019). Associations of meal timing and frequency with obesity and metabolic syndrome among Korean adults. Nutrients.

[103] Yoshida J., Eguchi E., Nagaoka K., Ito T., Ogino K. (2018). Association of night eating habits with metabolic syndrome and its components: A longitudinal study. BMC Public Health.

[104] Nishi T., Babazono A., Maeda T., Imatoh T., Une H. (2016). Effects of eating fast and eating before bedtime on the development of nonalcoholic fatty liver disease. Popul. Health Manag..

[105] Colles S.L., Dixon J.B., O’Brien P.E. (2007). Night eating syndrome and nocturnal snacking: Association with obesity, binge eating and psychological distress. Int. J. Obes..

[106] Kant A.K., Schatzkin A., Graubard B.I., Ballard-Barbash R. (1995). Frequency of eating occasions and weight change in the nhanes i epidemiologic follow-up study. Int. J. Obes. Relat. Metab. Disord..

[107] Coakley E.H., Rimm E.B., Colditz G., Kawachi I., Willett W. (1998). Predictors of weight change in men: Results from the health professionals follow-up study. Int. J. Obes. Relat. Metab. Disord..

[108] Leech R.M., Timperio A., Livingstone K.M., Worsley A., McNaughton S.A. (2017). Temporal eating patterns: Associations with nutrient intakes, diet quality, and measures of adiposity. Am. J. Clin. Nutr..

[109] Murakami K., Livingstone M.B. (2015). Eating frequency is positively associated with overweight and central obesity in U.S. Adults. J. Nutr..

[110] Halkjaer J., Tjonneland A., Overvad K., Sorensen T.I. (2009). Dietary predictors of 5-year changes in waist circumference. J. Am. Diet. Assoc..

[111] Blazey P., Habibi A., Hassen N., Friedman D., Khan K.M., Ardern C.L. (2023). The effects of eating frequency on changes in body composition and cardiometabolic health in adults: A systematic review with meta-analysis of randomized trials. Int. J. Behav. Nutr. Phys. Act..

[112] Kahleova H., Lloren J.I., Mashchak A., Hill M., Fraser G.E. (2017). Meal frequency and timing are associated with changes in body mass index in adventist health study 2. J. Nutr..

[113] Longo-Silva G., Lima M.O., Pedrosa A.K.P., Serenini R., Marinho P.M., Menezes R.C.E. (2024). Association of largest meal timing and eating frequency with body mass index and obesity. Clin. Nutr. ESPEN.

[114] Miyake T., Kumagi T., Hirooka M., Furukawa S., Kawasaki K., Koizumi M., Todo Y., Yamamoto S., Nunoi H., Tokumoto Y. (2015). Significance of exercise in nonalcoholic fatty liver disease in men: A community-based large cross-sectional study. J. Gastroenterol..

[115] Maruyama K., Sato S., Ohira T., Maeda K., Noda H., Kubota Y., Nishimura S., Kitamura A., Kiyama M., Okada T. (2008). The joint impact on being overweight of self reported behaviours of eating quickly and eating until full: Cross sectional survey. BMJ.

[116] Tsumura H., Fukuda M., Hisamatsu T., Sato R., Tsuchie R., Kanda H. (2023). Relationships of rapid eating with visceral and subcutaneous fat mass and plasma adiponectin concentration. Sci. Rep..

[117] Nishitani N., Sakakibara H., Akiyama I. (2009). Eating behavior related to obesity and job stress in male Japanese workers. Nutrition.

[118] Otsuka R., Tamakoshi K., Yatsuya H., Murata C., Sekiya A., Wada K., Zhang H.M., Matsushita K., Sugiura K., Takefuji S. (2006). Eating fast leads to obesity: Findings based on self-administered questionnaires among middle-aged Japanese men and women. J. Epidemiol..

[119] Zhang M., Sun X., Zhu X., Zheng L., Bi Y., Li Q., Sun L., Di F., Xu Y., Zhu D. (2024). Association between fast eating speed and metabolic dysfunction-associated steatotic liver disease: A multicenter cross-sectional study and meta-analysis. Nutr. Diabetes.

[120] Mansour-Ghanaei R., Mansour-Ghanaei F., Naghipour M., Joukar F. (2019). The lifestyle characteristics in non-alcoholic fatty liver disease in the persian guilan cohort study. Open Access Maced. J. Med. Sci..

[121] Lee S., Ko B.J., Gong Y., Han K., Lee A., Han B.D., Yoon Y.J., Park S., Kim J.H., Mantzoros C.S. (2016). Self-reported eating speed in relation to non-alcoholic fatty liver disease in adults. Eur. J. Nutr..

[122] Takahashi F., Hashimoto Y., Kawano R., Kaji A., Sakai R., Kawate Y., Okamura T., Ushigome E., Kitagawa N., Majima S. (2020). Eating fast is associated with nonalcoholic fatty liver disease in men but not in women with type 2 diabetes: A cross-sectional study. Nutrients.

[123] Cao X., Gu Y., Bian S., Zhang Q., Meng G., Liu L., Wu H., Zhang S., Wang Y., Zhang T. (2020). Association between eating speed and newly diagnosed nonalcoholic fatty liver disease among the general population. Nutr. Res..

[124] Kokkinos A., le Roux C.W., Alexiadou K., Tentolouris N., Vincent R.P., Kyriaki D., Perrea D., Ghatei M.A., Bloom S.R., Katsilambros N. (2010). Eating slowly increases the postprandial response of the anorexigenic gut hormones, peptide yy and glucagon-like peptide-1. J. Clin. Endocrinol. Metab..

[125] Zarrinpar A., Chaix A., Yooseph S., Panda S. (2014). Diet and feeding pattern affect the diurnal dynamics of the gut microbiome. Cell Metab..

[126] Thaiss C.A., Zeevi D., Levy M., Zilberman-Schapira G., Suez J., Tengeler A.C., Abramson L., Katz M.N., Korem T., Zmora N. (2014). Transkingdom control of microbiota diurnal oscillations promotes metabolic homeostasis. Cell.

[127] Teichman E.M., O’Riordan K.J., Gahan C.G.M., Dinan T.G., Cryan J.F. (2020). When rhythms meet the blues: Circadian interactions with the microbiota-gut-brain axis. Cell Metab..

[128] Cui Y., Li S., Yin Y., Li X., Li X. (2022). Daytime restricted feeding promotes circadian desynchrony and metabolic disruption with changes in bile acids profiles and gut microbiota in c57bl/6 male mice. J. Nutr. Biochem..

[129] Paukkonen I., Torronen E.N., Lok J., Schwab U., El-Nezami H. (2024). The impact of intermittent fasting on gut microbiota: A systematic review of human studies. Front. Nutr..

[130] Zeb F., Wu X., Chen L., Fatima S., Haq I.U., Chen A., Majeed F., Feng Q., Li M. (2020). Effect of time-restricted feeding on metabolic risk and circadian rhythm associated with gut microbiome in healthy males. Br. J. Nutr..

[131] Guo Y., Luo S., Ye Y., Yin S., Fan J., Xia M. (2021). Intermittent fasting improves cardiometabolic risk factors and alters gut microbiota in metabolic syndrome patients. J. Clin. Endocrinol. Metab..

[132] Ali I., Liu K., Long D., Faisal S., Hilal M.G., Ali I., Huang X., Long R. (2021). Ramadan fasting leads to shifts in human gut microbiota structured by dietary composition. Front. Microbiol..

[133] Su J., Wang Y., Zhang X., Ma M., Xie Z., Pan Q., Ma Z., Peppelenbosch M.P. (2021). Remodeling of the gut microbiome during ramadan-associated intermittent fasting. Am. J. Clin. Nutr..

[134] Cignarella F., Cantoni C., Ghezzi L., Salter A., Dorsett Y., Chen L., Phillips D., Weinstock G.M., Fontana L., Cross A.H. (2018). Intermittent fasting confers protection in cns autoimmunity by altering the gut microbiota. Cell Metab..

[135] Gabel K., Marcell J., Cares K., Kalam F., Cienfuegos S., Ezpeleta M., Varady K.A. (2020). Effect of time restricted feeding on the gut microbiome in adults with obesity: A pilot study. Nutr. Health.

[136] Ozkul C., Yalinay M., Karakan T. (2020). Structural changes in gut microbiome after ramadan fasting: A pilot study. Benef. Microbes.

[137] Singh A., Anjum B., Naz Q., Raza S., Sinha R.A., Ahmad M.K., Mehdi A.A., Verma N. (2024). Night shift-induced circadian disruption: Links to initiation of non-alcoholic fatty liver disease/non-alcoholic steatohepatitis and risk of hepatic cancer. Hepatoma Res..

[138] Fonken L.K., Workman J.L., Walton J.C., Weil Z.M., Morris J.S., Haim A., Nelson R.J. (2010). Light at night increases body mass by shifting the time of food intake. Proc. Natl. Acad. Sci. USA.

[139] Fonken L.K., Aubrecht T.G., Melendez-Fernandez O.H., Weil Z.M., Nelson R.J. (2013). Dim light at night disrupts molecular circadian rhythms and increases body weight. J. Biol. Rhythms.

[140] Schwartz W.J., Tavakoli-Nezhad M., Lambert C.M., Weaver D.R., de la Iglesia H.O. (2011). Distinct patterns of period gene expression in the suprachiasmatic nucleus underlie circadian clock photoentrainment by advances or delays. Proc. Natl. Acad. Sci. USA.

[141] Touitou Y., Point S. (2020). Effects and mechanisms of action of light-emitting diodes on the human retina and internal clock. Environ. Res..

[142] Baek J.H., Zhu Y., Jackson C.L., Park Y.M. (2024). Artificial light at night and type 2 diabetes mellitus. Diabetes Metab. J..

[143] Gradisar M., Wolfson A.R., Harvey A.G., Hale L., Rosenberg R., Czeisler C.A. (2013). The sleep and technology use of americans: Findings from the national sleep foundation’s 2011 sleep in america poll. J. Clin. Sleep Med..

[144] Falchi F., Cinzano P., Duriscoe D., Kyba C.C., Elvidge C.D., Baugh K., Portnov B.A., Rybnikova N.A., Furgoni R. (2016). The new world atlas of artificial night sky brightness. Sci. Adv..

[145] Obayashi K., Saeki K., Iwamoto J., Okamoto N., Tomioka K., Nezu S., Ikada Y., Kurumatani N. (2013). Exposure to light at night, nocturnal urinary melatonin excretion, and obesity/dyslipidemia in the elderly: A cross-sectional analysis of the heijo-kyo study. J. Clin. Endocrinol. Metab..

[146] Kim M., Vu T.H., Maas M.B., Braun R.I., Wolf M.S., Roenneberg T., Daviglus M.L., Reid K.J., Zee P.C. (2023). Light at night in older age is associated with obesity, diabetes, and hypertension. Sleep.

[147] Albreiki M.S., Middleton B., Hampton S.M. (2017). A single night light exposure acutely alters hormonal and metabolic responses in healthy participants. Endocr. Connect..

[148] Xu Y.X., Yu Y., Huang Y., Wan Y.H., Su P.Y., Tao F.B., Sun Y. (2022). Exposure to bedroom light pollution and cardiometabolic risk: A cohort study from chinese young adults. Environ. Pollut..

[149] Mason I.C., Grimaldi D., Reid K.J., Warlick C.D., Malkani R.G., Abbott S.M., Zee P.C. (2022). Light exposure during sleep impairs cardiometabolic function. Proc. Natl. Acad. Sci. USA.

[150] Windred D.P., Burns A.C., Rutter M.K., Ching Yeung C.H., Lane J.M., Xiao Q., Saxena R., Cain S.W., Phillips A.J.K. (2024). Personal light exposure patterns and incidence of type 2 diabetes: Analysis of 13 million hours of light sensor data and 670,000 person-years of prospective observation. Lancet Reg. Health Eur..

[151] Obayashi K., Saeki K., Iwamoto J., Ikada Y., Kurumatani N. (2014). Independent associations of exposure to evening light and nocturnal urinary melatonin excretion with diabetes in the elderly. Chronobiol. Int..

[152] Obayashi K., Yamagami Y., Kurumatani N., Saeki K. (2020). Bedroom lighting environment and incident diabetes mellitus: A longitudinal study of the heijo-kyo cohort. Sleep Med..

[153] Windred D.P., Burns A.C., Lane J.M., Olivier P., Rutter M.K., Saxena R., Phillips A.J.K., Cain S.W. (2024). Brighter nights and darker days predict higher mortality risk: A prospective analysis of personal light exposure in >88,000 individuals. Proc. Natl. Acad. Sci. USA.

[154] Zheng R., Xin Z., Li M., Wang T., Xu M., Lu J., Dai M., Zhang D., Chen Y., Wang S. (2023). Outdoor light at night in relation to glucose homoeostasis and diabetes in chinese adults: A national and cross-sectional study of 98,658 participants from 162 study sites. Diabetologia.

[155] Sorensen T.B., Wilson R., Gregson J., Shankar B., Dangour A.D., Kinra S. (2020). Is night-time light intensity associated with cardiovascular disease risk factors among adults in early-stage urbanisation in South India? A cross-sectional study of the andhra pradesh children and parents study. BMJ Open.

[156] Xu Z., Jin J., Yang T., Wang Y., Huang J., Pan X., Frank K., Li G. (2023). Outdoor light at night, genetic predisposition and type 2 diabetes mellitus: A prospective cohort study. Environ. Res..

[157] Vizcaino M., Buman M., DesRoches T., Wharton C. (2020). From tvs to tablets: The relation between device-specific screen time and health-related behaviors and characteristics. BMC Public Health.

[158] Padilla J., Osman N.M., Bissig-Choisat B., Grimm S.L., Qin X., Major A.M., Yang L., Lopez-Terrada D., Coarfa C., Li F. (2024). Circadian dysfunction induces nafld-related human liver cancer in a mouse model. J. Hepatol..

[159] Filipski E., Levi F. (2009). Circadian disruption in experimental cancer processes. Integr. Cancer Ther..

[160] Janse van Rensburg D.C., Jansen van Rensburg A., Fowler P.M., Bender A.M., Stevens D., Sullivan K.O., Fullagar H.H.K., Alonso J.M., Biggins M., Claassen-Smithers A. (2021). Managing travel fatigue and jet lag in athletes: A review and consensus statement. Sports Med..

[161] Boivin D.B., Boudreau P., Kosmadopoulos A. (2022). Disturbance of the circadian system in shift work and its health impact. J. Biol. Rhythms.

[162] Bonnefond A., Tassi P., Roge J., Muzet A. (2004). A critical review of techniques aiming at enhancing and sustaining worker’s alertness during the night shift. Ind. Health.

[163] Bescos R., Boden M.J., Jackson M.L., Trewin A.J., Marin E.C., Levinger I., Garnham A., Hiam D.S., Falcao-Tebas F., Conte F. (2018). Four days of simulated shift work reduces insulin sensitivity in humans. Acta Physiol..

[164] Wefers J., van Moorsel D., Hansen J., Connell N.J., Havekes B., Hoeks J., van Marken Lichtenbelt W.D., Duez H., Phielix E., Kalsbeek A. (2018). Circadian misalignment induces fatty acid metabolism gene profiles and compromises insulin sensitivity in human skeletal muscle. Proc. Natl. Acad. Sci. USA.

[165] Van Cauter E., Desir D., Decoster C., Fery F., Balasse E.O. (1989). Nocturnal decrease in glucose tolerance during constant glucose infusion. J. Clin. Endocrinol. Metab..

[166] Sun M., Feng W., Wang F., Li P., Li Z., Li M., Tse G., Vlaanderen J., Vermeulen R., Tse L.A. (2018). Meta-analysis on shift work and risks of specific obesity types. Obes. Rev..

[167] Watanabe K., Sakuraya A., Kawakami N., Imamura K., Ando E., Asai Y., Eguchi H., Kobayashi Y., Nishida N., Arima H. (2018). Work-related psychosocial factors and metabolic syndrome onset among workers: A systematic review and meta-analysis. Obes. Rev..

[168] Li W., Chen Z., Ruan W., Yi G., Wang D., Lu Z. (2019). A meta-analysis of cohort studies including dose-response relationship between shift work and the risk of diabetes mellitus. Eur. J. Epidemiol..

[169] Vetter C., Dashti H.S., Lane J.M., Anderson S.G., Schernhammer E.S., Rutter M.K., Saxena R., Scheer F. (2018). Night shift work, genetic risk, and type 2 diabetes in the UK biobank. Diabetes Care.

[170] van Duijne H.M., Berentzen N.E., Vermeulen R.C.H., Vlaanderen J.J., Kromhout H., Jozwiak K., Pijpe A., Rookus M.A., van Leeuwen F.E., Schaapveld M. (2024). Associations of night shift work with weight gain among female nurses in the Netherlands: Results of a prospective cohort study. Scand. J. Work. Environ. Health.

[171] Pan A., Schernhammer E.S., Sun Q., Hu F.B. (2011). Rotating night shift work and risk of type 2 diabetes: Two prospective cohort studies in women. PLoS Med..

[172] Manodpitipong A., Saetung S., Nimitphong H., Siwasaranond N., Wongphan T., Sornsiriwong C., Luckanajantachote P., Mangjit P., Keesukphan P., Crowley S.J. (2017). Night-shift work is associated with poorer glycaemic control in patients with type 2 diabetes. J. Sleep Res..

[173] Zhang S., Wang Y., Wang Z., Wang H., Xue C., Li Q., Guan W., Yuan J. (2020). Rotating night shift work and non-alcoholic fatty liver disease among steelworkers in china: A cross-sectional survey. Occup. Environ. Med..

[174] Maidstone R., Rutter M.K., Marjot T., Ray D.W., Baxter M. (2024). Shift work and evening chronotype are associated with hepatic fat fraction and non-alcoholic fatty liver disease in 282,303 UK biobank participants. Endocr. Connect..

[175] Huang H., Liu Z., Xie J., Xu C. (2023). Association between night shift work and nafld: A prospective analysis of 281,280 uk biobank participants. BMC Public Health.

[176] Lee Y., Lee W. (2024). Shift work and non-alcoholic fatty liver disease in young, healthy workers. Sci. Rep..

[177] Xu J., Ni S., Wang Y., Yan M., Yang X., Ge H., Jia Z., Yang Z., Shan A., Liu H. (2023). Shift work and nonalcoholic fatty liver disease incidence among chinese rail workers: A 4-year longitudinal cohort study. Int. Arch. Occup. Environ. Health.

[178] Kim K., Lee Y.J., Kwon S.C., Min Y.S., Lee H.K., Baek G., Kim S.H., Jang E.C. (2022). Correlation between shift work and non-alcoholic fatty liver disease among male workers in the steel manufacturing company of korea: A cross-sectional study. Ann. Occup. Environ. Med..

[179] Balakrishnan M., El-Serag H.B., Kanwal F., Thrift A.P. (2017). Shiftwork is not associated with increased risk of nafld: Findings from the national health and nutrition examination survey. Dig. Dis. Sci..

[180] Zhang S., Wang Y., Zhu Y., Li X., Song Y., Yuan J. (2020). Rotating night shift work, exposure to light at night, and glomerular filtration rate: Baseline results from a chinese occupational cohort. Int. J. Environ. Res. Public Health.

[181] Chang Q., Zhu Y., Liang H., Cheng J., Li D., Lin F., Zhou X., Pan P., Ma F., Zhang Y. (2024). Night shift work associates with all-cause and cause-specific mortality: A large prospective cohort study. J. Gen. Intern. Med..

[182] Manoogian E.N.C., Zadourian A., Lo H.C., Gutierrez N.R., Shoghi A., Rosander A., Pazargadi A., Ormiston C.K., Wang X., Sui J. (2022). Feasibility of time-restricted eating and impacts on cardiometabolic health in 24-h shift workers: The healthy heroes randomized control trial. Cell Metab..

[183] Zhu Y., Liu J., Jiang H., Brown T.J., Tian Q., Yang Y., Wang C., Xu H., Liu J., Gan Y. (2020). Are long working hours associated with weight-related outcomes? A meta-analysis of observational studies. Obes. Rev..

[184] Baek S.U., Won J.U., Lee Y.M., Yoon J.H. (2024). Association between long working hours and metabolic dysfunction-associated steatotic liver disease: A nationwide population-based study in Korea. Public Health.

[185] Song J.H., Kim H.R., Lee D.W., Min J., Lee Y.M., Kang M.Y. (2022). Association between long working hours and liver enzymes: Evidence from the korea national health and nutrition examination survey, 2007–2017. Ann. Occup. Environ. Med..

[186] Song E., Kim J.A., Roh E., Yu J.H., Kim N.H., Yoo H.J., Seo J.A., Kim S.G., Kim N.H., Baik S.H. (2021). Long working hours and risk of nonalcoholic fatty liver disease: Korea national health and nutrition examination survey vii. Front. Endocrinol..

[187] Kivimaki M., Jokela M., Nyberg S.T., Singh-Manoux A., Fransson E.I., Alfredsson L., Bjorner J.B., Borritz M., Burr H., Casini A. (2015). Long working hours and risk of coronary heart disease and stroke: A systematic review and meta-analysis of published and unpublished data for 603,838 individuals. Lancet.

[188] Qiu D., Li Y., Li R., He J., Ouyang F., Luo D., Xiao S. (2022). Long working hours, work-related stressors and sleep disturbances among Chinese government employees: A large population-based follow-up study. Sleep Med..

[189] Schneider S., Becker S. (2005). Prevalence of physical activity among the working population and correlation with work-related factors: Results from the first german national health survey. J. Occup. Health.

[190] Min J., Lee D.W., Kang M.Y., Myong J.P., Kim H.R., Lee J. (2022). Working for long hours is associated with dietary fiber insufficiency. Front. Nutr..

[191] Oostenbach L.H., Lamb K.E., Crawford D., Thornton L. (2022). Influence of work hours and commute time on food practices: A longitudinal analysis of the household, income and labour dynamics in australia survey. BMJ Open.

[192] Tanaka R., Tsuji M., Kusuhara K., Kawamoto T., Japan E., Children’s Study G. (2018). Association between time-related work factors and dietary behaviors: Results from the Japan environment and children’s study (jecs). Environ. Health Prev. Med..

[193] Blafoss R., Micheletti J.K., Sundstrup E., Jakobsen M.D., Bay H., Andersen L.L. (2019). Is fatigue after work a barrier for leisure-time physical activity? Cross-sectional study among 10,000 adults from the general working population. Scand. J. Public Health.

[194] Kim C., Jin H., Dusing G.J. (2023). Employment conditions and leisure-time physical activity among Korean workers: A longitudinal study (2009–2019). BMC Public Health.

[195] Maruyama S., Morimoto K. (1996). Effects of long workhours on life-style, stress and quality of life among intermediate Japanese managers. Scand. J. Work. Environ. Health.

[196] Ekiz Erim S., Sert H. (2023). The relationship between chronotype and obesity: A systematic review. Chronobiol. Int..

[197] Yu J.H., Yun C.H., Ahn J.H., Suh S., Cho H.J., Lee S.K., Yoo H.J., Seo J.A., Kim S.G., Choi K.M. (2015). Evening chronotype is associated with metabolic disorders and body composition in middle-aged adults. J. Clin. Endocrinol. Metab..

[198] Merikanto I., Lahti T., Puolijoki H., Vanhala M., Peltonen M., Laatikainen T., Vartiainen E., Salomaa V., Kronholm E., Partonen T. (2013). Associations of chronotype and sleep with cardiovascular diseases and type 2 diabetes. Chronobiol. Int..

[199] Hu C., Zhang Y., Wang S., Lin L., Peng K., Du R., Qi H., Zhang J., Wang T., Zhao Z. (2020). Association of bedtime with the risk of non-alcoholic fatty liver disease among middle-aged and elderly chinese adults with pre-diabetes and diabetes. Diabetes Metab. Res. Rev..

[200] Wang H., Gu Y., Zheng L., Liu L., Meng G., Wu H., Xia Y., Bao X., Shi H., Sun S. (2018). Association between bedtime and the prevalence of newly diagnosed non-alcoholic fatty liver disease in adults. Liver Int..

[201] Weng Z., Ou W., Huang J., Singh M., Wang M., Zhu Y., Kumar R., Lin S. (2021). Circadian misalignment rather than sleep duration is associated with mafld: A population-based propensity score-matched study. Nat. Sci. Sleep.

[202] Spiegel K., Leproult R., Van Cauter E. (1999). Impact of sleep debt on metabolic and endocrine function. Lancet.

[203] Chaput J.P. (2014). Sleep patterns, diet quality and energy balance. Physiol. Behav..

[204] Olds T.S., Maher C.A., Matricciani L. (2011). Sleep duration or bedtime? Exploring the relationship between sleep habits and weight status and activity patterns. Sleep.

[205] Maukonen M., Kanerva N., Partonen T., Kronholm E., Tapanainen H., Kontto J., Mannisto S. (2017). Chronotype differences in timing of energy and macronutrient intakes: A population-based study in adults. Obesity.

[206] Baron K.G., Reid K.J., Kern A.S., Zee P.C. (2011). Role of sleep timing in caloric intake and bmi. Obesity.

[207] VoPham T., Weaver M.D., Vetter C., Hart J.E., Tamimi R.M., Laden F., Bertrand K.A. (2018). Circadian misalignment and hepatocellular carcinoma incidence in the United States. Cancer Epidemiol. Biomarkers Prev..

[208] Yang J., Zhang K., Xi Z., Ma Y., Shao C., Wang W., Tang Y.D. (2023). Short sleep duration and the risk of nonalcoholic fatty liver disease/metabolic associated fatty liver disease: A systematic review and meta-analysis. Sleep Breath..

[209] Shen N., Wang P., Yan W. (2016). Sleep duration and the risk of fatty liver disease: A systematic review and meta-analysis. Sci. Rep..

[210] Wijarnpreecha K., Thongprayoon C., Panjawatanan P., Ungprasert P. (2016). Short sleep duration and risk of nonalcoholic fatty liver disease: A systematic review and meta-analysis. J. Gastroenterol. Hepatol..

[211] Zong G., Mao W., Wen M., Cheng X., Liu G. (2024). Association of sleep patterns and disorders with metabolic dysfunction-associated steatotic liver disease and liver fibrosis in contemporary American adults. Ann. Hepatol..

[212] Suikki T., Maukonen M., Partonen T., Jousilahti P., Kanerva N., Mannisto S. (2021). Association between social jet lag, quality of diet and obesity by diurnal preference in finnish adult population. Chronobiol. Int..

[213] Islam Z., Akter S., Kochi T., Hu H., Eguchi M., Yamaguchi M., Kuwahara K., Kabe I., Mizoue T. (2018). Association of social jetlag with metabolic syndrome among Japanese working population: The furukawa nutrition and health study. Sleep Med..

[214] Koopman A.D.M., Rauh S.P., van’t Riet E., Groeneveld L., van der Heijden A.A., Elders P.J., Dekker J.M., Nijpels G., Beulens J.W., Rutters F. (2017). The association between social jetlag, the metabolic syndrome, and type 2 diabetes mellitus in the general population: The new hoorn study. J. Biol. Rhythms.

[215] Anothaisintawee T., Lertrattananon D., Thamakaison S., Thakkinstian A., Reutrakul S. (2018). The relationship among morningness-eveningness, sleep duration, social jetlag, and body mass index in asian patients with prediabetes. Front. Endocrinol..

[216] Gao L., Gong J., Zhong G., Qin Y. (2024). Day napping and metabolic-associated fatty liver disease: A systematic review and meta-analysis of observational studies. Medicine.

[217] Yang X., Wang J., Wang H. (2023). Association between sleep traits and primary liver cancer: A mendelian randomization analysis. Eur. J. Clin. Investig..

[218] Woods D.L., Kim H., Yefimova M. (2013). To nap or not to nap: Excessive daytime napping is associated with elevated evening cortisol in nursing home residents with dementia. Biol. Res. Nurs..

[219] Stergiou G.S., Mastorantonakis S.E., Roussias L.G. (2008). Intraindividual reproducibility of blood pressure surge upon rising after nighttime sleep and siesta. Hypertens. Res..

[220] Hu L.Y., Chen P.M., Hu Y.W., Shen C.C., Perng C.L., Su T.P., Yen S.H., Tzeng C.H., Chiou T.J., Yeh C.M. (2013). The risk of cancer among patients with sleep disturbance: A nationwide retrospective study in Taiwan. Ann. Epidemiol..

